# Beyond competencies – describing work ready plus graduates for the New Zealand medical imaging workforce

**DOI:** 10.1002/jmrs.290

**Published:** 2018-07-01

**Authors:** Dale Sheehan, Tracy M. de Bueger, Joanna Thorogood, Sharon Sitters, Arun Deo

**Affiliations:** ^1^ Gold Coast University Hospital Gold Coast Queensland Australia; ^2^ Unitec Auckland New Zealand; ^3^ Waitemata District Health Board Auckland New Zealand

**Keywords:** Education, general, medical imaging, professional

## Abstract

**Introduction:**

Medical imaging is a profession where technology and practice is moving forward at a fast pace, while also being situated within rapidly evolving models of healthcare delivery. This requires capabilities in our graduates beyond the competencies to undertake the role. This study used an existing Professional Capability Framework as the foundation for the development of a survey tool to identify those capabilities most valued by the health services that provide placement opportunities for students.

**Methods:**

A prospective survey, conducted online, was used to gather the data from senior medical imaging staff who work regularly with students and new graduates.

**Results:**

The results identified the top capabilities within each of three domains of personal, interpersonal, and cognitive capabilities. The results from the personal domain show that remaining calm under pressure or when things take an unexpected turn; understanding personal strengths and limitations; being willing to face and learn from errors; wanting to do as good a job as possible and having energy, passion and enthusiasm for the profession and role are most valued by the profession. We have identified links between the most desired interpersonal capabilities and attributes of well‐functioning interprofessional teams and the cognitive capabilities align to those required for critical thinking and clinical reasoning.

**Conclusions:**

These results have allowed the curriculum development team to review the graduate profile of medical imaging technology graduates. The curriculum development team has begun a process to incorporate learning strategies that will encourage the development of these capabilities in our graduates.

## Introduction

There is a growing interest globally in making sure that graduates emerge from higher education with the capabilities and competencies that will equip them not only to be ‘work ready’ on graduation but also prepared for the development of technology, new models of service delivery and advances for practice in the future.^1^, ^2^, ^3^ In a profession, such as medical imaging, the health workforce needs graduates who are ready to understand and apply emerging technology alongside meeting the demands of ever changing healthcare systems.^4^

This paper reports on the outcomes of a survey undertaken as part of preparation for the review and redesign of clinical placements in a medical imaging programme in New Zealand. The project embraced the goal of defining work ready plus graduates for the medical imaging workforce. Identification of the capabilities required of a medical imaging technologist (MIT) in their graduate years was critical for the development of the clinical experience programme, as it is clinical placement and emersion in work that is most likely to develop capability and work readiness skills in graduates. It was envisaged that by defining, for our regional context, the capabilities and work skills employers seek in our graduates, we would have the data we needed to review and if necessary rewrite the graduate profile and utilise fully and effectively the real‐life clinical experiences that support the development of these capabilities. The results are also impacting positively on lecturers teaching methods as they consider how they can develop these capabilities in students through teaching, learning, and assessment methodologies.

The theoretical underpinning for this study was Scott's fellowship work for the Australian Teaching and Learning Council and the professional and graduate capability framework published for the Australian tertiary environment.^1^ The Professional Capability Framework as used by Western Sydney University was used as the foundation for the development of a survey tool as it was current and had been validated in a range of disciplines that included health professions. In addition, it looks beyond graduation and standards for practice (as required by the New Zealand Medical Radiation Technologist Registration Board and the Medical Radiation Practice Board of Australia towards the generic skills graduates need to flourish in a profession in the future.^1^, ^5^ Hence the term work ready plus. Using a validated and comprehensive professional and graduate capability framework ensured that all potentially relevant capability options had been considered. It was deemed generalisable to the New Zealand health care environment due to the similarities between both the health and education systems.

**Figure 1:**
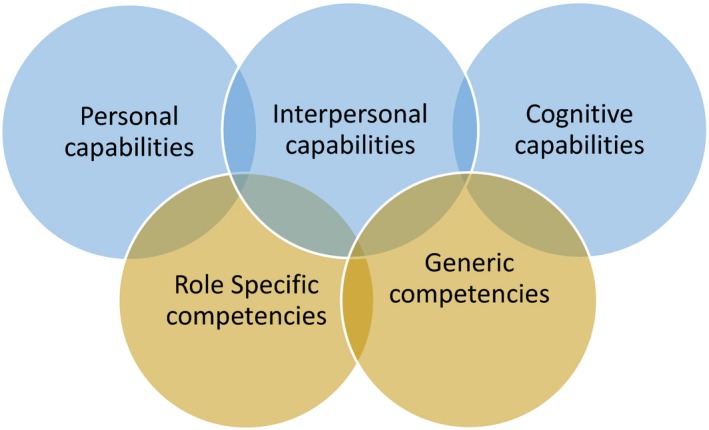
Professional capability framework.^1^ Permission was obtained to reproduce this figure.

Figure 1 summarises the key elements of the professional capability framework. The overlapping aspects of professional capability are identified – personal, interpersonal and cognitive which have been validated in a range of investigations, mainly focused on professional leadership.^1^, ^5^ These domains are underpinned by relevant role‐specific and generic competencies (the skills and knowledge found to be essential to the specific role of an MIT). The key terms “competence” and “capability” are problematic and therefore often confused. We adopted the definition that competence is the possession of the skills and knowledge necessary to perform the duties set down for a specific role. The New Zealand Medical Radiation Technologist Registration Board (MRTB) reviewed and updated their competencies for New Zealand registration in March 2017, so a list of competencies was current and available. We have adopted a definition of capability that goes beyond the skills to practice as a safe and competent practitioner, to embrace the concept of being work ready plus. Being “work ready *plus”* requires capabilities for not just today, for current practice but for the future. Capabilities include the ability to work with others from a range of professions and backgrounds, manage the unexpected, adopt new technology, to be changed implementation savvy, inventive, sustainability responsive, to learn from experience and to operate with a clear understanding of one's ethical position.^1^

These capabilities require a mixture of emotional and cognitive intelligence, including the ability to determine when and when not to deploy these competences.^1^ We believed this concept was less developed for the medical imaging profession in New Zealand.

The Professional Capability Framework developed through a scholarship awarded by the Australian Teaching and Learning Council formed the basis for the development of a survey that asked practicing MITs and MIT clinical managers at the three largest placements sites in New Zealand to rate the capabilities deemed critical in a graduate to ensure they are “work ready *plus”*.^1^

The items used in the survey fall into three domains which align with the capability domains identified in Figure 1. These domains are discussed in more detail in Scott, Coates and Anderson^5^ and Fullan and Scott.^6^

This paper shares the results from the survey and discusses the impact these are having on curriculum review and development.

## Method

This study was carried within all the public (three District Health Board, which includes 2 hospitals on the Northshore, 3 Inner City and 1 in South Auckland), Radiology Services, in the Auckland Region, where Unitec Institute of Technology's MIT students are placed for clinical experience during their 3‐year training programme. Data collection period, April and August 2017.

A prospective survey was selected as the method of data collection tool as it allowed us to collect anonymous responses from stakeholders with minimal disruption to the work environment. The survey was distributed electronically.

The SurveyMonkey online tool was used to develop a rating scale questionnaire, using the statements and domains from the Australian Capability Framework.^1^

The survey was trialled by three clinicians and during this process one question was removed that was perceived repetitive. The final questionnaire had 39 capability statements that were clustered into three domains: personal, interpersonal and cognitive. The first question of the questionnaire requested participants consent before proceeding with the survey.

An open survey link was sent to MIT clinical managers for internal circulation. A participant information sheet was attached to the email invitation email. Participants were assured of the anonymity of their responses and this was achieved by using the anonymity function on SurveyMonkey.

Ethics approval was granted by the Unitec Research Ethics Committee (UREC) – No 2017–1002.

### Analysis design

For the demographic variables of the survey, the data were represented either in the form of tables or graphs.

Owing to the subjective nature of the data related to capabilities that participants were requested to provide in ordinal form (ranking), the average ranking measure was considered most appropriate to statistically determine which answer choice was most preferred overall. The answer choice with the largest average ranking is the most preferred choice.

The calculations were conducted using Microsoft Excel. The questionnaire was organised with a total of 39 statements which were grouped into the three domain categories: personal capabilities included 15 statements, interpersonal capabilities had 11 statements and cognitive capabilities had 13 statements. Thus, the ranking for personal capabilities was from 1 to 15, interpersonal from 1 to 11 and cognitive from 1 to 13.

The average ranking was calculated as follows:

$$\mathrm{Average}\;\mathrm{Ranking}=\frac{x_{1}w_{1}+x_{2}w_{2}+\ldots +x_{n}w_{n}}{\mathrm{Total}},$$

where *w* represented the weight of ranked position and *x* represented the response count for the answer choice.

Weights are applied in reverse order. The respondent's most preferred choice, which is ranked 1, has the largest weight and their least preferred choice has a weight of 1. In our case, the personal capabilities had 15 statements. The highest ranked statement had a weight of 15, second highest had 14, third highest had 13 and so on with the last ranked statement having a weight of 1. Similar weights, depending on the number of statements, were applied to the interpersonal and cognitive capabilities.

## Results

A total of 52 responses were received from a maximum sample size of 265. This indicates a response rate of 19.6%. However, it is not possible to exactly predict the size of the actual sample pool, as the surveys were distributed via the clinical managers to their staff. From the responses, 90% (47) of the respondents were female and the remaining 10% (5) were males. In terms of the position/title of the respondents, the majority of the respondents (76%) were senior qualified MITs and 15% team leader/clinical specialist. 74% (39) had over 6 years experience.

### Average ranking reported by domain

Table 1 shows the average rankings for the domain personal capabilities with the top five clearly visible.

**Table 1:** Average ranking scores for personal capabilities

Statements – personal capabilities	Average ranking score
Being willing to face and learn from errors	11.56
Wanting to do as good a job as possible	11.21
Understanding personal strengths and limitations	11.12
Remaining calm under pressure or when things take an unexpected turn	10.98
Having energy, passion and enthusiasm for the profession and role	10.94
Willingness to persevere when things are not working out as anticipated	8.35
Pitching in and undertaking menial tasks as required	7.44
Being true to one's personal values and ethics	7.27
Deferring judgment and not jumping in too quickly to resolve a problem	7.1
Maintaining a good work/life balance and keeping things in perspective	6.61
Being willing to take a hard decision	6.52
Bouncing back from adversity	6.37
Being confident to take calculated risks	5.71
Being willing to take responsibility for projects and how they turn out	5.62
Tolerating ambiguity and uncertainty	4.69

Table 2 shows the average rankings for the domain interpersonal capabilities. These rankings have a flatter profile with eight capabilities ranking higher than 5.

**Table 2:** Average ranking scores for interpersonal capabilities

Statements – interpersonal capabilities	Average ranking score
Being transparent and honest in dealings with others	7.64
Empathising and working productively with people from a wide range of backgrounds	7.57
Listening to different points of view before coming to a decision	7.13
Understanding how the different groups that make up a work place operate and influence different situations	7.02
Giving and receiving constructive feedback to/from work colleagues and others	6.73
Being able to develop and contribute positively to team‐based programs	6.1
Being able to work with senior staff within and beyond the organisation without being intimidated	5.87
Motivating others to achieve positive outcomes	5.85
Being able to develop and use networks of colleagues to solve key workplace problems	4.77
Influencing people's behaviour and decisions in effective ways	3.96
Working constructively with people who are ‘resistors’ or are over‐enthusiastic	3.58

Table 3 shows the average rankings for the domain cognitive capabilities. All cognitive capabilities achieved an average ranking score of more than 5.

**Table 3:** Average ranking scores for cognitive capabilities

Statements – cognitive capabilities	Average ranking score
Diagnosing the underlying causes of a problem and taking appropriate action to address it	9.2
Making sense of and learning from experience	9.19
Being able to identify the core issue from a mass of detail in any situation	8.04
Using previous experience to figure out what's going on when a current situation takes an unexpected turn	7.96
Having a clear, justified and achievable direction in area of responsibility	7.85
Thinking creatively and laterally	6.92
Seeing the best way to respond to a perplexing situation	6.72
Setting and justifying priorities for daily work	6.71
Adjusting a plan of action in response to problems that are identified during its implementation	6.67
Recognising patterns in a complex situation	5.94
Seeing and then acting on an opportunity for a new direction	5.88
Recognising how seemingly unconnected activities are linked	5.23
Tracing out and assessing the likely consequences of alternative courses of action	5.15

## Discussion

### Personal domain capabilities

The results from the personal domain show a clear top five capability rated highly by the respondents. Namely: remaining calm under pressure or when things take an unexpected turn; understanding personal strengths and limitations; being willing to face and learn from errors; wanting to do as good a job as possible; having energy, passion and enthusiasm for the profession and role. These five capabilities had strong face validity when presented to a meeting of national managers. They certainly provide a clear mandate as to which personal qualities should be incorporated into the graduate profile. The challenge in curriculum design will be to find ways to highlight, reinforce and role model these capabilities. The literature provides limited guidance, however Fraser & Greenhalgh^3^ suggest that capability can be strengthened by the use of feedback, self‐reflection, and consolidation, with students following a nonlinear education model. Therefore, the incorporation of directed educator and supervisor feedback could assist in the recognition and development of this capability. To further consolidate these skills, it would be advantageous to encourage students to observe these capabilities in others and reflect as to how their own developing practice incorporates and builds this capability.

### Interpersonal domain capabilities

We have noted that in the interpersonal domain, the six top ranked qualities (all with a ranking above 6) have an alignment to the competencies identified for those working in interprofessional teams. Ponzer et al.^7^ published the five core competencies that form the basis of many interprofessional education activities which have been modified for specific contexts and are frequently used to describe strong interprofessional teams.^8^ Table 4 compares these.

**Table 4:** Frequently used statements of attributes of effective interprofessional teams compared to the top six interprofessional capabilities in this study

Interpersonal capabilities compared to interprofessional team attributes	
This study's highest ranked interpersonal capabilities	Interprofessional team attributes
Understanding how the different groups that make up a work place operate and influence different situations Empathising and working productively with people from a wide range of backgrounds Being able to develop and contribute positively to team‐based programs Giving and receiving constructive feedback to/from work colleagues and others Listening to different points of view before coming to a decision Being transparent and honest in dealings	Mutual understanding of roles and recognition of difference Good patient‐ care/co‐operation Mutual trust and respect The importance of good communication for teamwork Assertiveness needed for effective conflict management Be aware of ethical issues

Following the World Health Organisation report in 2010^9^ there is a growing international commitment to the promotion of collaborative practice in healthcare delivery supported by interprofessional education to ensure graduates have the capabilities required for collaborative roles on graduation. In 2012, the national boards and the Australian Health Ministers’ Advisory Council, conducted an independent Review of the National Registration and Accreditation Scheme for health professionals.^10^ As part of this review, the Health Practitioner Regulation Agency (AHPRA) reviewed the performance of each of these accreditation authorities against the domains of the Quality Framework for the Accreditation Function to inform the decisions on how to continue to implement the accreditation function under the National Law. Following this review process, all the current profession‐specific accreditation authorities were asked to consider opportunities to increase cross‐profession collaboration and innovation and support interprofessional learning.^10^ There is a close working relationship between New Zealand and Australian registration and accrediting bodies and considerable influence in both directions. The growth of the Australian interprofessional agenda is likely to have a growing impact on New Zealand health professional registration and accreditation requirements. The capabilities for team work and collaborative practice recognised by practicing MITs in New Zealand appears to support the growing international agenda supporting collaborative practice models of care.

There has been some substantial work in the interprofessional education and team working space around both learning and assessment methods that we can use to guide our curriculum planning.^11^, ^12^, ^13^ Simulation with other professionals, interprofessional activities within the academic curriculum and opportunities to observe and engage with interprofessional teams while on placement (evidenced in a clinical portfolio) align to these capabilities.

### Cognitive domain capabilities

In the cognitive abilities domain diagnosing underlying causes of a problem, taking appropriate action and making sense of learning from experience are the most highly rated, followed by being able to identify the core issue from a mass of detail in any situation and using previous experience to figure out what's going on when a current situation takes an unexpected turn, are capabilities that aid problem solving. Overall the profile of preference in this domain is relatively flat. We note alignment to the concepts of clinical reasoning and critical thinking as it is described in the health professions. In the literature, the terms clinical reasoning, clinical judgment, problem‐solving, decision‐making and critical thinking are often used interchangeably. The term clinical reasoning is used to describe the process by which clinicians collect cues, process the information, come to an understanding of a patient problem or situation, plan and implement interventions, evaluate outcomes, and reflect on and learn from the process.^14^, ^15^, ^16^ The clinical reasoning process is also described as dependent upon a critical thinking “disposition”.^17^ The American Philosophical Association defined critical thinking as purposeful, self‐regulatory judgment that uses cognitive tools such as interpretation, analysis, evaluation, inference, and explanation of the evidential, conceptual, methodological, criteriological or contextual considerations on which judgment is based.^18^ We have noted that some students have a problem understanding how these capabilities are demonstrated in the work place. Responses in this domain are assisting us to define what capabilities are associated with critical thinking and clinical reasoning in the medical imaging profession and how they are evidenced in clinical practice. We are turning our attention to building processes to support the development of these capabilities within our class‐based learning, simulated learning, and clinical supervision. We will also incorporate post‐practicum experiences that will encourage students to appraise their experiences, seek clarification and comparisons and link their learning to the future, including securing employment.^19^ The goal is to develop the student's ability to make judgements and decisions about their work experiences and learning that will position them as future critical thinkers, life longer enquirers and learners.

## Conclusion

Identification of the core capabilities that our stakeholder community rate highly has proved informative in assisting us to describe a “work ready *plus”* medical imaging graduate for the New Zealand context. The results have provided data to the curriculum development team allowing them to align the graduate profile to these expectations and raised awareness among academic staff of the need to include these capabilities in the curriculum. In addition, it has enabled a dialog with stakeholders about capability in the profession, refreshing and revising the involvement of the professional community in the academic programme.

Scott reminds us that capability cannot be taught, people cannot be trained in it; but it can be learnt through exposure to educational experiences which entail coming to grips with real world dilemmas. Clinical placements provide this learning experience; it is here students learn what others do when the unexpected happens and develop the skills to make sense of what is unfolding to successfully resolve the situation. This naturally occurring curriculum of the workplace is often tacit and therefore not clearly visible to learners and students needs support.^19^ These results provide a blue print for conceptualising the key opportunities a clinical placement offers beyond learning technical skills and competencies; highlighting the capabilities that can be learnt and developed on placement, bringing these learning opportunities to the attention of students and clinical supervisors alike and bringing a new clarity to the design of support for learning on placement. We now have descriptors of capability that will allow us to be more specific in our communication of the capabilities our graduates should aspire to (beyond but building on those established by the regulatory body) and we are incorporating these into the curriculum design process for both teaching and assessment purposes. They will inform clinical supervision and clinical learning, allowing clinical supervisors to focus on highlighting experiences that can develop these capabilities.

This study is informing curriculum planning and energising discussions around the design of simulation, class room teaching activities and clinical placements designed to develop these capabilities.

## Conflict of Interest

The authors declare no conflict of interest.
